# The epidemiology, antifungal use and risk factors of death in elderly patients with candidemia: a multicentre retrospective study

**DOI:** 10.1186/s12879-014-0609-x

**Published:** 2014-11-25

**Authors:** Hao Wang, Naizheng Liu, Mei Yin, Hui Han, Jinfeng Yue, Fan Zhang, Tichao Shan, Haipeng Guo, Dawei Wu

**Affiliations:** Department of Critical Care Medicine, Qilu Hospital, Shandong University, 107 Wenhua-xi Road, Jinan, 250012 Shandong China; Department of Emergency, Liaocheng People’s Hospital affiliated with Taishan Medical College, 67 Dongchang-xi Road, Liaocheng, 252000 Shandong, China; Department of Geriatrics, Qilu Hospital, Shandong University, 107 Wenhua-xi Road, Jinan, 250012 Shandong China

**Keywords:** Candidemia, Elderly, Antifungal agent, Risk factors, Death

## Abstract

**Background:**

The elderly patients affected by candidemia are growing in proportion to inpatients, but available data are limited. We aimed to determine the epidemiology, antifungal management and clinical risk factors of death in the elderly population with candidemia in China.

**Methods:**

This retrospective study included 63 elderly (≥65 years) and 84 younger patients (16–60 years) at 4 tertiary hospitals. Multivariable logistic regression model was used to identify independent risk factors of death in elderly patients.

**Results:**

The distribution of *Candida* species did not differ between elderly and younger patients (*p* >0.05). Resistance to fluconazole and voriconazole for non-*Candida albicans* species in elderly patients was approximately double that in younger patients. Host-related risk factors (e.g., underlying solid tumour, diabetes mellitus and chronic renal failure) and hospital-related factors (e.g., prior stay in an intensive care unit, mechanical ventilation, central vascular and urethral catheters placement) were identified more common in elderly patients. Elderly patients less often received triazoles and were less likely to receive antifungal therapies mostly because elderly or their guardians quit antifungal therapies. APACHE II scores and 30-day mortality were higher for elderly than younger patients (31.7% vs. 16.7%, *p* =0.032). For elderly patients, antifungal therapy administered before microbiological documentation was the only protective factor for death, whereas absence of antifungal therapies, receipt of mechanical ventilation and APACHE II score ≥20 were independent predictors of death.

**Conclusions:**

Elderly patients with candidemia had poor prognoses characterized by certain host and hospital-related risk factors and special pathogen resistance features. More awareness of the burden of this disease is required, and the absence of antifungal therapies should be avoided to improve the prognoses of elderly patients with this severe infection.

## Background

Candidemia has emerged as an important nosocomial infection associated with significant morbidity and mortality [1]-[3]. It is the fourth most common nosocomial bloodstream infection in the United States and the seventh in Europe [4],[5]. It prolongs hospital stays and increases the costs associated with patient management [6]. The epidemiology of candidemia has been studied extensively worldwide, and data are available from a large series of laboratory-based and population-based surveillance studies, as well as studies focusing on specific patient populations such as neonates and those with cancer, undergoing surgery, and staying in intensive care units (ICUs) patients [7]-[11].

The elderly population is large and is growing in proportion to the general hospitalized population. Because of comorbidities, aging and age-associated physiological changes, increased rates of oropharyngeal colonization with *Candida* species, and concomitant drug use, elderly patients are more vulnerable to *Candida* infections [12],[13]. The features of the pathogen, risk factors and severity of candidemia in elderly patients may differ from that in younger adults and might be associated with worse prognosis [14]. Moreover, physicians may choose different antifungal management for elderly and younger patients, depending on patient’ compliance, individual experiences and different guidelines [15]. However, available data for elderly patients with candidemia are limited [16],[17].

Here, we hypothesized that candidemia in elderly patients would present several peculiarities in epidemiology, clinical risk factors for death and antifungal management. To characterize these peculiarities, we evaluated a database of patients with candidemia from 4 multicentre, retrospective surveillance studies coordinated by our group, and compared the above indicators in elderly versus younger patients. Furthermore, to improve the clinical management and outcome of elderly patients, we used multiple logistic analyses to identify the risk factors for death.

## Methods

### Study design and patient selection

We performed a multicentre retrospective study of candidemia in 4 tertiary general hospitals in Shandong, China: Qilu Hospital of Shandong University (Jinan, 2000 beds), Qianfoshan Hospital affiliated with Shandong University (Jinan, 1500 beds), Jinan Center Hospital affiliated with Shandong University (Jinan, 1000 beds), and Liaocheng People’s Hospital affiliated with Taishan Medical College (Liaocheng, 2000 beds). The study was conducted from June 2008 to June 2010. We included patients ≥16 years who had hospital acquired candidemia*,* which was defined as at least one positive blood culture for *Candida* species in patients hospitalized for more than 48 hrs with clinical signs and symptoms of sepsis [18]. Elderly patients were defined as the patients 65 years of age or older, whereas patients with the age between 16 and 60 were defined as younger patients. All episodes of candidemia were identified via the laboratory computer system. For patients with multiple candidemic episodes, only the first episode was included. Patients with candidemia caused simultaneously by different species of *Candida* were excluded. The study was approved by the Ethics Committee of Qilu Hospital of Shandong University and was carried out according to the ethical standards set forth in the Declaration of Helsinki of 1964.

### Data collection

Trained study team members collected demographic and clinical data by chart review. Demographic and microbiological data, underlying diseases, predisposing factors, laboratory data, concurrent infections, antifungal agents exposure and outcome were recorded on standardized case report forms.

Candida species were identified by use of the VITEK-32 system (BioMerieux Vitek, St. Louis, MO, USA). We used the recently updated species-specific antifungal drug susceptibility thresholds for fluconazole, voriconazole and caspofungin by the Clinical and Laboratory Standards Institute (CLSI) [19]-[21]. Isolates of *C. krusei* were considered intrinsically resistant to fluconazole. For voriconazole, isolates of *C. glabrata* and *C. krusei* isolates with minimal inhibitory concentration (MIC) of ≥2 μg/ml were considered resistant. Predisposing factors (including immunosuppressive drugs and severe hypoalbuminemia) and underlying diseases (including solid organ tumour, hematologic malignancy, neutropenia, diabetes mellitus and chronic renal failure) were recorded as host-related risk factors [22]. Other predisposing factors, including surgeries, abdominal surgical operations, ICU stay ≥5 days, multiple blood product transfusion, parenteral nutrition, receipt of mechanical ventilation, placement of central venous catheter (CVC) or urethral catheter, and prior antibacterial exposure were recorded as hospital-related risk factors [22]. All predisposing factors had to occur within 30 days before the onset of candidemia. CVC-related candidemia required the isolation of the same *Candida* spp. from both blood and catheter tip.

The severity of the initial presentation of candidemia was assessed by the Acute Physiology and Chronic Health Evaluation (APACHE) II score [23]. Because of the specific purpose of the study, we additionally reported an age-adjusted APACHE II score in which points attributed to older age were subtracted from the total score. We recorded the antifungal agents used for more than 2 days within 2 weeks before and 2 weeks after the microbiological documentation of candidemia.

Neutropenia was defined as absolute neutrophil count <0.5 × 10^9^/l. Severe hypoalbuminemia was defined as serum albumin levels <23 g/L. Immunosuppressive drugs received included glucocorticoids (≥20 mg/day of prednisone or equivalent doses of other corticosteroids for >1 week), chemotherapy drugs or other immunosuppressive agents. Multiple blood product transfusion was defined as transfusion of ≥6 units consisting of at least 2 units of erythrocytes and 2 units of fresh frozen plasma. Septic shock was defined as systolic blood pressure <90 mmHg, diastolic blood pressure <60 mmHg, or fluid/inotrope required to maintain blood pressure above these levels. Concurrent bloodstream infections were defined as an isolation of a positive culture for bacterium in patients with signs or symptoms of infection that occurred within 2 weeks of the onset of candidemia.

### Statistical analyses

The data for categorical variables are expressed as percentages and continuous variables as mean ± SD or median with inter-quartile range (IQR). Chi-square or Fisher’s exact test (two-tailed) was used to compare categorical variables and unpaired Student’s *t* test or Mann–Whitney *U* test to compare continuous variables. Multivariable, backwards, stepwise, logistic regression analyses was used to identify independent risk factors associated with day-30 mortality of elderly patients with candidemia. Variables with *p* ≤0.10 on univariate analyses were entered into the multivariable model. Statistical analyses involved use of SPSS v15.0 for Windows (SPSS Inc*.*, Chicago, IL, USA). A *p* <0.05 was considered statistically significant.

## Results

### Patient demographics

We detected 154 cases of candidemia and excluded 7 cases of polyfungal candidemia, leaving 147 cases in 147 patients for analyses (mean age 55.0 ± 12.4 years; 110 men): 63 elderly patients (42.9%) and 84 (57.1%) younger patients. The 2 groups did not differ in male sex and length of stay (Table 1). The elderly patients were more often admitted to ICUs than younger patients (49.2% vs. 23.8%; *p =*0.001) and were less often admitted to medical wards (22.2% vs. 38.1%; *p* = 0.040).

**Table 1:** **Demographics and clinical risk factors of the elderly and younger patients with candidemia**

Characteristics	Elderly patients (≥65 years, *n* =63)	Younger patients (16–60 years, *n* =84)	*p* value
Age (years)^a^	75.4 ± 12.7	39.6 ± 11.2	<0.001
Male sex	51 (81.0)	59 (70.2)	0.139
Length of stay^a^	45.3 ± 32.8	39.2 ± 30.9	0.251
Hospital settings			
ICUs	31 (49.2)	20 (23.8)	0.001
Medical wards	14 (22.2)	32 (38.1)	0.040
Surgical wards	12 (19.0)	25 (29.8)	0.139
Others	6 (9.5)	7 (8.3)	0.801
Host-related risk factors			
Solid tumour	15 (23.8)	5 (6.0)	0.002
Haematological malignancy^b^	3 (4.8)	14 (16.7)	0.026
Neutropenia	5 (7.9)	20 (23.8)	0.011
Diabetes mellitus	24 (38.1)	8 (9.5)	0.001
Chronic renal failure	18 (28.6)	7 (8.3)	0.001
Prior immunosuppressive drugs	16 (25.4)	25 (29.8)	0.559
Severe hypoalbuminemia	13 (20.6)	10 (11.9)	0.149
Hospital-related risk factors			
Surgery	14 (22.2)	29 (34.5)	0.105
Abdominal surgical operations	9 (14.3)	14 (16.7)	0.694
ICU stay ≥5 days	19 (30.2)	6 (7.1)	0.001
Multiple blood product transfusion	8 (12.7)	16 (19.0)	0.303
Parental nutrition	20 (31.7)	16 (19.0)	0.076
Receipt of mechanical ventilation	27 (42.9)	20 (23.8)	0.014
CVC placement	32 (50.8)	26 (31.0)	0.015
Urethral catheter placement	34 (54.0)	29 (34.5)	0.018
Prior antibacterial exposure^b^	63 (100.0)	82 (97.6)	0.607
≥ 3 kinds of prior antibacterial drugs	29 (46.0)	34 (40.5)	0.501

Data are *n* (%) or mean ± SD.

ICU, intensive care unit; CVC, central venous catheter.

^a^Two-independent samples *t*-test; ^b^Fisher’s exact test; Unspecified: chi-square test.

### Microbiology of *Candida*species

The *Candida* species isolated were as follows: 27 *C. albicans* (42.9%), 16 *C. tropicalis* (25.4%), 9 *C. parapsilosis* (14.3%), 7 *C. glabrata* (11.1%), 3 *C. krusei* (4.8%) and 1 *C. famata* (1.6%) in the elderly patients; 31 *C. albicans* (36.9%), 19 *C. tropicalis* (22.6%), 17 *C. parapsilosis* (20.2%), 9 *C. glabrata* (10.7%), 6 *C. krusei* (7.1%), and *C. rugosa* and *C. guilliermondii* (*n =* 1 each; 1.2%) in younger patients (Table 2). The distribution of *Candida* species did not differ between elderly and younger patients (*p* >0.05).

**Table 2:** ***Candida*** **species distribution and resistance to antifungal agents isolated from patients**

*Candida* species	Elderly patients (≥65 years, *n* =63)	Younger patients (16-60 years, *n* =84)
***Candida albicans***	27/63 (42.9)	31/84 (36.9)
Fluconazole	1/27 (3.7)	1/31 (3.2)
Voriconazole	1/27 (3.7)	0/31 (0)
Caspofungin	0/27 (0)	0/31 (0)
**Non-** ***C. albicans*** **species**	36/63 (57.1)	53/84 (63.1)
Fluconazole	11/36 (30.6)	8/53 (15.1)
Voriconazole	3/36 (8.3)	2/53 (3.8)
Caspofungin	0/36 (0)	0/53 (0)
*Candida tropicalis*	16/63 (25.4)	19/84 (22.6)
Fluconazole	4/16 (25.0)	1/19 (5.3)
Voriconazole	2/16 (12.5)	1/19 (5.3)
Caspofungin	0/16 (0)	0/19 (0)
*Candida parapsilosis*	9/63 (14.3)	17/84 (20.2)
Fluconazole	2/9 (22.2)	1/17 (5.9)
Voriconazole	0/9 (0.0)	0/17 (0)
Caspofungin	0/9 (0)	0/17 (0)
*Candida glabrata*	7/63 (11.1)	9/84 (10.7)
Fluconazole	2/7 (28.6)	0/9 (0)
Voriconazole	1/7 (14.3)	0/9 (0)
Caspofungin	0/7 (0)	0/9 (0)
*Candida krusei* ^a^	3/63 (4.8)	6/84 (7.1)
Fluconazole	3/3 (100.0)	6/6 (100.0)
Voriconazole	0/3 (0)	1/6 (16.7%)
Caspofungin	0/3 (0)	0/6 (0)
Other^a^	1/63 (1.6)^*^	2/84 (2.4) ^†^
Fluconazole	0/1 (0)	0/2 (0)
Voriconazole	0/1 (0)	0/2 (0)
Caspofungin	0/1 (0)	0/2 (0)

Data are *n* (%).

^*^including 1 *C. famat.*

^†^including 1 *C. rugosa* and 1 *C. guilliermondii*.

We found resistance to fluconazole, voriconazole and caspofungin for *C. albicans* in 1/27 (3.7%), 1/27 (3.7%), 0/27 (0%) in elderly patients and 1/31 (3.2%), 0/31 (0%), 0/31 (0%) in younger patients, respectively (Table 2). In elderly patients, the resistance to fluconazole and voriconazole of non-*C. albicans* species was approximate double that of younger patients [30.6% (11 of 36) vs. 15.1% (8 of 53), 8.3% (3 of 36) vs. 3.8% (2 of 53), respectively]; however, all the isolates in younger and elderly patients were susceptible to caspofungin.

### Clinical risk factors

Elderly patients more often presented with solid tumour (23.8% vs. 6.0%; *p =*0.002), diabetes mellitus (38.1% vs. 9.5%; *p =*0.001), or chronic renal failure (28.6% vs. 8.3%; *p =*0.001), and less often with haematological malignancy (16.7% vs. 4.8%; *p =*0.026) and neutropenia (23.8% vs. 7.9%; *p =*0.011). In addition, elderly patients more often underwent procedures defined as hospital-related risk factors, including prior ICU stay ≥5 days (30.2% vs. 7.1%; *p =*0.001), mechanical ventilation (42.9% vs. 23.8%; *p =*0.014), CVC (50.8% vs. 31.0%; *p =*0.015) and urethral catheter placement (54.0% vs. 34.5%; *p =*0.018) (Table 1). The incidence of the other risk factors, including prior immunosuppressive drugs and severe hypoalbuminemia, abdominal surgical operations, multiple blood product transfusion, parental nutrition and prior antibacterial exposure, did not differ between the two groups.

### Antifungal agent exposure

The 2 age groups did not differ in the selection of antifungal agents before or after the identification of *Candida* species (all *p* >0.05) (Table 3). Overall, elderly patients were less often administered triazoles (57.1% vs. 78.6%; *p* =0.005) and were more often lacking antifungal therapies (25.4% vs. 11.9%; *p* =0.034) compared with younger patients (Table 3). The possible reasons for the absence of antifungal therapy in the elderly were determined from the medical records: 11 patients or their guardians decided to quit antifungal therapy, mainly because of hopelessness in the recovery, high hospitalization expenses, or non-coverage of antifungal agents by medical insurance; 2 patients died before microbiologicy documentation of *Candida* in the bloodstream; and 3 patients with undetermined reasons.

**Table 3:** **Antifungal therapy started before and after microbiological documentation of** ***Candida*** **in the bloodstream**

Characteristics	Elderly patients (≥65 years, *n* =63)	Younger patients (16-60 years, *n* =84)	*p* value
Antifungal therapy administered before microbiological documentation	22 (34.9%)	47 (56.0%)	0.011
Treatment method			
Prophylactic treatment	7 (11.1%)	17 (20.2%)	0.138
Empiric treatment	12 (19.0%)	23 (27.4%)	0.240
Undetermined^a^	3 (4.8%)	7 (8.3%)	0.603
Agents selection			
Fluconazole	16 (25.4%)	32 (38.1%)	0.104
Itraconazole	3 (4.8%)	11 (13.1%)	0.089
Voriconazole^a^	3 (4.8%)	4 (4.8%)	0.696
Antifungal therapy administered/changed after microbiological documentation	28 (44.4%)	36 (42.9%)	0.848
Agents selection			
Fluconazole	4 (6.3%)	13 (15.5%)	0.087
Itraconazole^a^	6 (9.5%)	3 (3.6%)	0.253
Voriconazole^a^	4 (6.3%)	6 (7.1%)	0.887
Micafungin^a^	3 (4.8%)	5 (6.0%)	0.958
Caspofungin	11 (17.5%)	9 (10.7%)	0.238
Overall			
Triazoles usage	36 (57.1%)	66 (78.6%)	0.005
Echinocandins usage	14 (22.2%)	14 (16.7%)	0.396
Absence of antifungal therapies	16 (25.4%)	10 (11.9%)	0.034

Data are presented as *n* (%).

Antifungal agents were changed in 3 elderly patients and 6 younger patients.

^a^Fisher’s exact test; Unspecified: chi-square test.

### Laboratory data, outcomes and risk factors for death in elderly patients

Compared with younger patients, elderly patients were sicker, as shown by significant higher serum creatinine level (112.0 ± 95.3 vs. 82.4 ± 65.6, *p* =0.027) and blood sugar level (9.3 ± 5.1 vs. 6.7 ± 2.8, *p* =0.001) as well as significantly higher APACHE II score (20.3 ± 8.1 vs. 14.6 ± 7.3; *p* =0.001) (Table 4). However, no significant differences were noted in age-adjusted APACHE II scores (16.2 ± 7.7 vs. 13.9 ± 7.1; *p* =0.063). The elderly patients had significantly higher 30-day mortality rates (31.7% vs. 16.7%, *p* =0.032).

**Table 4:** **Laboratory data and outcomes of the elderly and younger patients with candidemia**

Characteristics	Elderly patients (≥65 years, *n* =63)	Younger patients (16–60 years, *n* =84)	*p* value
Laboratory data			
Hemoglobin level (g/L)	86.9 ± 29.1	94.2 ± 30.9	0.148
Platelet count (×10^9^/L)	163.4 ± 93.7	153.6 ± 117.1	0.586
Serum creatinine level ( mol/L)	112.0 ± 95.3	82.4 ± 65.6	0.027
Blood sugar level (mmol/L)	9.3 ± 5.1	6.7 ± 2.8	0.001
Serum sodium level (mmol/L)	138.6 ± 5.8	137.5 ± 5.4	0.238
Serum potassium level (mmol/L)	3.9 ± 1.0	3.8 ± 0.9	0.526
Total bilirubin level ( mol/L)^b^	16.3 (6.2-31.8)	13.9 (8.3-30.6)	0.271
High fever (>39°C)^a^	19 (30.2)	32 (38.1)	0.317
Removal of CVC^a^	14/32 (43.8%)	15/26 (57.7)	0.291
CVC-related candidemia^a^	8 (12.7%)	10 (11.9%)	0.885
Concurrent bloodstream infection^a^	8 (12.7)	14 (16.7)	0.505
APACHE II score	20.3 ± 8.1	14.6 ± 7.3	0.001
Age-adjusted APACHE II score	16.2 ± 7.7	13.9 ± 7.1	0.063
Septic shock^a^	27 (42.9)	24 (28.6)	0.072
30-day mortality rate^a^	20 (31.7)	14 (16.7)	0.032

Data are *n* (%), mean ± SD or median (IQR).

APACHE, Acute Physiology and Chronic Health Evaluation; CVC, central venous catheter.

^a^Chi-square test; ^b^Mann-Whitney *U* test; Unspecified: Two-independent samples *t*-test.

On univariate analyses, compared with non-survivors, survivors showed higher, although not significantly proportion of prophylactic and empiric treatment (all *p* >0.10) (Table 5); the rate of CVC removal was higher for survivors than non- survivors (*p* <0.10) and was included in the multivariable regression analyses. On multivariable analyses, antifungal therapy administered before microbiological documentation was the only protective factor for death (odds ratio [OR]0.8, 95% confidence interval [95% CI] 0.7–0.9, *p* =0.046). Independent predictors of death were absence of antifungal therapies (OR 2.1; 95% CI 1.2–23.8; *p* =0.039), receipt of mechanical ventilation (OR 3.5; 95% CI 1.6–12.4; *p* =0.042) and APACHE II score ≥20 (OR 4.0; 95% CI 2.5–8.6; *p* =0.018).

**Table 5:** **Univariate analysis of outcome in elderly patients with candidemia**

Characteristics	Non-survivors ( *n* =20)	Survivors ( *n* =43)	*p* value
Age ≥85 years^a^	4 (20.0)	5 (11.6)	0.619
Male sex	17 (85.0)	34 (79.1)	0.831
Solid tumor^a^	8 (40.0)	7 (16.3)	0.082
Diabetes mellitus	8 (40.0)	16 (37.2)	0.832
Chronic renal failure	9 (45.0)	9 (20.9)	0.049
Prior immunosuppressive drugs	5 (25.0)	11 (25.6)	0.961
Severe hypoalbuminemia	8 (40.0)	5 (11.6)	0.040
Surgery^a^	5 (25.0)	9 (20.9)	0.971
ICU stay ≥5 days	9 (45.0)	10 (23.3)	0.080
Parental nutrition	7 (35.0)	13 (30.2)	0.705
Receipt of mechanical ventilation	14 (70.0)	13 (30.2)	0.003
CVC placement	9 (45.0)	23 (53.5)	0.530
CVC-related candidemia^a^	1 (5.0)	7 (16.3)	0.398
CVC removal^a^	1 (5.0)	13 (30.2)	0.055
Antifungal therapy administered before microbiological documentation	3 (15.0)	19 (44.2)	0.024
Prophylactic treatment^a^	1 (5.0)	6 (14.0)	0.534
Empiric treatment^a^	2 (10.0)	10 (23.3)	0.367
Antifungal therapy administered/changed after microbiological documentation	8 (40.0)	20 (46.5)	0.628
Triazoles usage	9 (45.0)	27 (62.8)	0.184
Echinocandins usage ^a^	2 (10.0)	12 (27.9)	0.206
Absence of antifungal therapies	10 (50.0)	6 (14.0)	0.002
Platelet count ×100 g/L × 10^9^/L	7 (35.0)	5 (11.6)	0.028
Serum creatinine level ≥180 μmol/L	8 (40.0)	7 (16.3)	0.040
Total bilirubin level ≥30 μmol/L	9 (45.0)	7 (16.3)	0.015
Concurrent bacteraemia	6 (30.0)	2 (4.7)	0.016
APACHE II score ≥20	15 (75.0)	16 (37.2)	0.005
Septic shock	13 (65.0)	14 (32.6)	0.015

Data are *n* (%).

ICU, intensive care unit; CVC, central venous catheter; APACHE, Acute Physiology and Chronic Health Evaluation.

^a^Fisher’s exact test; Unspecified: chi-square test.

## Discussion

A major strength of this study is that our data are representative of 4 centres and included patients from different hospital settings in China. The resistance to fluconazole and vorizonazole of non-*C. albicans* species in elderly patients being double that in younger patients should be addressed when applying empirical or prophylactic antifungal therapy. As well, risk factors related to host characteristics (e.g., underlying solid tumour, diabetes mellitus and chronic renal failure) and hospital exposure (e.g., prior ICU stay, mechanical ventilation, central venous catheters and urethral catheter placement) were identified more commonly in elderly patients than younger patients. Thirdly, as a independent risk factor for death in elderly, the absence of antifungal therapies was more common than younger patients, mostly because the patients or their guardians decided to quit antifungal therapy.

The rapidly growing elderly population has specific physiological characteristics, which makes it susceptible to colonization and subsequent infection due to *Candida* species [24]. Not surprisingly, in this study, the prevalence of host-related risk factors, including solid tumour, diabetes mellitus and chronic renal failure, was greater for elderly than younger patients with candidemia. Cancer was a frequent underlying disease in both younger and older patients, but younger patients incurred a higher incidence of haematological malignancies, whereas elderly patients were more likely to present solid tumours, which is consistent with a previous report [25].

Another main feature in the diagnosis of candidemia is the evaluation of hospital-related risk factors [26]. Luzzati et al. [27] reported that candidemia in elderly patients was strongly associated with duration of total and peripheral parenteral nutrition, other central vascular catheters and glycopeptide antibiotics. Here, we identified more healthcare-related factors, including prior ICU stay, mechanical ventilation and urethral catheter placement in elderly patients. To decrease the incidence of candidemia in elderly patients, methods aimed to reduce unnecessary medical procedures should be encouraged whenever feasible.

The elderly patients are particularly susceptible to various infections and exposed to numerous antibiotic and antifungal treatments. Exposure to antibiotics and antifungal agents induces antifungal resistance and is an important cause of increased azoles-resistant *Candida* isolates [28]-[30], which might explain our finding of high resistance to azoles of non-*C. albicans* species in elderly patients. Non-*C. albicans* species such as *C. glabrata, C. parapsilosis* and *C. tropicalis* are especially vulnerable to acquiring resistance after a period of exposure to antifungal agent [31]. The emergence of fluconazole resistance in *C. parapsilosis* occurred after more than 10 years of fluconazole prophylaxis, which suggests that the use of fluconazole prophylaxis contributed to the emergence of *C. parapsilosis* with decreased susceptibility among the isolates responsible for bloodstream infections [32]. *C. glabrata* may be intermediately resistant to all azoles, and about 20% of strains develop resistance during therapy and prophylaxis with fluconazole [13]; previous fluconazole use is a significant risk factor for health care-associated fluconazole-resistant *C. glabrata* [33]. Considering the low resistance to caspofungin in the *Candida* isolates in elderly patients, caspofungin might be a better alternative to treat candidemia in this population.

In this study, elderly patients were more likely to experience septic shock and have poor outcomes than younger patients. The difference in outcome may be explained by higher severity of illness, as evidenced by increased APACHE II score, a widely recognized scoring system used to evaluate the severity of illness in critically ill patients [23]. The APACHE II score is calculated from 12 routine physiological measurements, including age, mean arterial pressure, heart rate, creatinine level, arterial pH, serum potassium and sodium levels, hematocrit value, Glasgow Coma Scale. However, after calculating an age-adjusted APACHE II score, we found no significant difference in illness severity between elderly and younger groups. Thus, advanced age rather than other indicators involved in the APACHE II score was the crucial factor determining the worse prognosis of elderly patients.

The elderly patients were more likely to not have antifungal treatment than younger patients, and in agreement with the previous report [34], the absence of antifungal agents was independently associated with worse prognosis in elderly. Therefore, prompt initiation of early antifungal therapy is warranted in high-risk elderly patients. Elderly patients or their guardians were likely to quit therapy when the patient’s conditions worsened, mainly because of lack of hope for the patient’s recovery, high hospitalization expenses, or non-coverage of antifungal agents by medical insurance. The poor care for elderly patients from family members and society, as well as the lag in the medical insurance industry, are challenges to the health of geriatric populations in China [35]. To decrease the high mortality of elderly patients with candidemia, the absence of antifungal therapy should be avoided by taking measures to correct the above causes.

One of main points regarding candidemia is that delaying antifungal treatment significantly increases mortality [36]. Early treatment strategies, including prophylactic and empiric treatment is beneficial for patients with candidemia and decrease mortality [34],[37]. Here, early antifungal treatment administered before microbiology documentation was a protective factor for death and could improve the outcome of elderly patients. However, we found no role for prophylactic and empiric treatment in the outcome of candidemia episodes: prophylactic and empiric treatment was used more often, although not significantly, for survivors than non-survivors, mostly because of the small number of older patients. A larger investigation with more cases is warranted to disclose the potential role of different treatment regimens on outcome in older population.

Our observations have several limitations. First the retrospective design of the study limited our ability to obtain exact variables such as prior antifungal exposures, which could be important for the emergence of antifungal resistance of non-*C. albicans* species. Second, compared to results from previous reports [38]-[40], the removal rate in the elderly patients was relatively low (43.8%), so the real proportion of CVC-related candidemia as well as the effect of CVC removal on prognosis might have been underestimated. Considering that CVC retention has a negative impact on outcome in patients with candidemia [38]-[40], the awareness of the risk of CVC retention needs to be strengthened in the management of candidemia in Chinese hospitals. Third, we considered only the presence or absence of risk factor exposure, not the duration of exposure. Because the study was not designed to quantify the length of exposure, this variable was not available for analyses, and its associated bias could not be determined. Furthermore, some of our conclusions may not be generalizable to other countries because of differences in antifungal usage and the epidemiology of candidemia. Further studies are necessary in different geographical areas.

## Conclusion

In conclusion, the elderly patients account for a substantial proportion of patients with candidemia and have higher mortality than younger patients. Such patients are characterized by certain host and hospital-related risk factors as well as special pathogen resistance features. More awareness of the burden of this disease is required and the absence of antifungal therapies should be avoided to improve the prognoses of elderly patients with this severe infection.

## Consent

Written informed consent was obtained from the patient for the publication of this report and any accompanying images.

## Authors’ contributions

HW participated in the conception and design, data analysis, data interpretation and manuscript writing. NL participated in the design of the study and data analysis. MY participated in the data analysis and interpretation. HH participated in the literature search, study design and data acquisition. JY and FZ participated in the data acquisition, data analysis and interpretation. TS participated in the data analysis and drafted the manuscript. HG participated in the design of the study and performed the statistical analysis. DW conceived of the study, and participated in its design and coordination and helped to draft the manuscript. All authors read and approved the final manuscript.

## References

[1] Zaoutis TE, Argon J, Chu J, Berlin JA, Walsh TJ, Feudtner C (2005). The epidemiology and attributable outcomes of candidemia in adults and children hospitalized in the United States: a propensity analysis. Clin Infect Dis.

[2] Horn DL, Neofytos D, Anaissie EJ, Fishman JA, Steinbach WJ, Olyaei AJ, Marr KA, Pfaller MA, Chang CH, Webster KM (2009). Epidemiology and outcomes of candidemia in 2019 patients: data from the prospective antifungal therapy alliance registry. Clin Infect Dis.

[3] Ostrosky-Zeichner L, Kullberg BJ, Bow EJ, Hadley S, Leon C, Nucci M, Patterson TF, Perfect JR (2011). Early treatment of candidemia in adults: a review. Med Mycol.

[4] Lamagni TL, Evans BG, Shigematsu M, Johnson EM (2001). Emerging trends in the epidemiology of invasive mycoses in England and Wales (1990-9). Epidemiol Infect.

[5] Wisplinghoff H, Bischoff T, Tallent SM, Seifert H, Wenzel RP, Edmond MB (2004). Nosocomial bloodstream infections in US hospitals: analysis of 24,179 cases from a prospective nationwide surveillance study. Clin Infect Dis.

[6] Gudlaugsson O, Gillespie S, Lee K, Vande Berg J, Hu J, Messer S, Herwaldt L, Pfaller M, Diekema D (2003). Attributable mortality of nosocomial candidemia, revisited. Clin Infect Dis.

[7] Leroy O, Gangneux JP, Montravers P, Mira JP, Gouin F, Sollet JP, Carlet J, Reynes J, Rosenheim M, Regnier B, Lortholary O (2009). Epidemiology, management, and risk factors for death of invasive Candida infections in critical care: a multicenter, prospective, observational study in France (2005-2006). Crit Care Med.

[8] Neu N, Malik M, Lunding A, Whittier S, Alba L, Kubin C, Saiman L (2009). Epidemiology of candidemia at a Children’s hospital, 2002 to 2006. Pediatr Infect Dis J.

[9] Pyrgos V, Ratanavanich K, Donegan N, Veis J, Walsh TJ, Shoham S (2009). Candida bloodstream infections in hemodialysis recipients. Med Mycol.

[10] Sipsas NV, Lewis RE, Tarrand J, Hachem R, Rolston KV, Raad II, Kontoyiannis DP (2009). Candidemia in patients with hematologic malignancies in the era of new antifungal agents (2001-2007): stable incidence but changing epidemiology of a still frequently lethal infection. Cancer.

[11] Benjamin DK, Stoll BJ, Fanaroff AA, McDonald SA, Oh W, Higgins RD, Duara S, Poole K, Laptook A, Goldberg R (2006). Neonatal candidiasis among extremely low birth weight infants: risk factors, mortality rates, and neurodevelopmental outcomes at 18 to 22 months. Pediatrics.

[12] Kauffman CA (2001). Fungal infections in older adults. Clin Infect Dis.

[13] Pfaller MA, Diekema DJ (2007). Epidemiology of invasive candidiasis: a persistent public health problem. Clin Microbiol Rev.

[14] Yoshikawa TT (2000). Epidemiology and unique aspects of aging and infectious diseases. Clin Infect Dis.

[15] Guery BP, Arendrup MC, Auzinger G, Azoulay E, Borges Sa M, Johnson EM, Muller E, Putensen C, Rotstein C, Sganga G, Venditti M, Zaragoza Crespo R, Kullberg BJ (2009). Management of invasive candidiasis and candidemia in adult non-neutropenic intensive care unit patients: Part II. Treatment. Intensive Care Med.

[16] Blot S, Cankurtaran M, Petrovic M, Vandijck D, Lizy C, Decruyenaere J, Danneels C, Vandewoude K, Piette A, Vershraegen G, Van Den Noortgate N, Peleman R, Vogelaers D (2009). Epidemiology and outcome of nosocomial bloodstream infection in elderly critically ill patients: a comparison between middle-aged, old, and very old patients. Crit Care Med.

[17] Dimopoulos G, Paiva JA, Meersseman W, Pachl J, Grigoras I, Sganga G, Montravers P, Auzinger G, Sa MB, Miller PJ, Marcek T, Kantecki M, Ruhnke M (2012). Efficacy and safety of anidulafungin in elderly, critically ill patients with invasive Candida infections: a post hoc analysis. Int J Antimicrob Agents.

[18] Annane D, Bellissant E, Cavaillon JM (2005). Septic shock. Lancet.

[19] Pfaller MA, Andes D, Diekema DJ, Espinel-Ingroff A, Sheehan D (2010). Wild-type MIC distributions, epidemiological cutoff values and species-specific clinical breakpoints for fluconazole and Candida: time for harmonization of CLSI and EUCAST broth microdilution methods. Drug Resist Updat.

[20] Pfaller MA, Diekema DJ, Andes D, Arendrup MC, Brown SD, Lockhart SR, Motyl M, Perlin DS (2011). Clinical breakpoints for the echinocandins and Candida revisited: integration of molecular, clinical, and microbiological data to arrive at species-specific interpretive criteria. Drug Resist Updat.

[21] Pfaller MA, Andes D, Arendrup MC, Diekema DJ, Espinel-Ingroff A, Alexander BD, Brown SD, Chaturvedi V, Fowler CL, Ghannoum MA, Johnson EM, Knapp CC, Motyl MR, Ostrosky-Zeichner L, Walsh TJ (2011). Clinical breakpoints for voriconazole and Candida spp. revisited: review of microbiologic, molecular, pharmacodynamic, and clinical data as they pertain to the development of species-specific interpretive criteria. Diagn Microbiol Infect Dis.

[22] Yapar N, Pullukcu H, Avkan-Oguz V, Sayin-Kutlu S, Ertugrul B, Sacar S, Cetin B, Kaya O (2011). Evaluation of species distribution and risk factors of candidemia: a multicenter case–control study. Med Mycol.

[23] Chang RW, Jacobs S, Lee B (1988). Predicting outcome among intensive care unit patients using computerised trend analysis of daily Apache II scores corrected for organ system failure. Intensive Care Med.

[24] Flevari A, Theodorakopoulou M, Velegraki A, Armaganidis A, Dimopoulos G (2013). Treatment of invasive candidiasis in the elderly: a review. J Clin Interv Aging.

[25] Guimaraes T, Nucci M, Mendonca JS, Martinez R, Brito LR, Silva N, Moretti ML, Salomao R, Colombo AL (2012). Epidemiology and predictors of a poor outcome in elderly patients with candidemia. Int J Infect Dis.

[26] Guery BP, Arendrup MC, Auzinger G, Azoulay E, Borges Sa M, Johnson EM, Muller E, Putensen C, Rotstein C, Sganga G, Venditti M, Zaragoza Crespo R, Kullberg BJ (2009). Management of invasive candidiasis and candidemia in adult non-neutropenic intensive care unit patients: Part I. Epidemiology and diagnosis. Intensive Care Med.

[27] Luzzati R, Cavinato S, Giangreco M, Grana G, Centonze S, Deiana ML, Biolo G, Barbone F (2013). Peripheral and total parenteral nutrition as the strongest risk factors for nosocomial candidemia in elderly patients: a matched case–control study. Mycoses.

[28] Ben-Ami R, Olshtain-Pops K, Krieger M, Oren I, Bishara J, Dan M, Wiener-Well Y, Weinberger M, Zimhony O, Chowers M, Weber G, Potasman I, Chazan B, Kassis I, Shalit I, Block C, Keller N, Kontoyiannis DP, Giladi M (2012). Antibiotic exposure as a risk factor for fluconazole-resistant Candida bloodstream infection. Antimicrob Agents Chemother.

[29] Garnacho-Montero J, Diaz-Martin A, Garcia-Cabrera E, Ruiz Perez de Pipaon M, Hernandez-Caballero C, Aznar-Martin J, Cisneros JM, Ortiz-Leyba C (2010). Risk factors for fluconazole-resistant candidemia. Antimicrob Agents Chemother.

[30] Joseph-Horne T, Hollomon DW (1997). Molecular mechanisms of azole resistance in fungi. FEMS Microbiol Lett.

[31] Silva S, Negri M, Henriques M, Oliveira R, Williams DW, Azeredo J (2012). Candida glabrata, Candida parapsilosis and Candida tropicalis: biology, epidemiology, pathogenicity and antifungal resistance. FEMS Microbiol Rev.

[32] Sarvikivi E, Lyytikainen O, Soll DR, Pujol C, Pfaller MA, Richardson M, Koukila-Kahkola P, Luukkainen P, Saxen H (2005). Emergence of fluconazole resistance in a Candida parapsilosis strain that caused infections in a neonatal intensive care unit. J Clin Microbiol.

[33] Lee I, Fishman NO, Zaoutis TE, Morales KH, Weiner MG, Synnestvedt M, Nachamkin I, Lautenbach E (2009). Risk factors for fluconazole-resistant Candida glabrata bloodstream infections. Arch Intern Med.

[34] Morrell M, Fraser VJ, Kollef MH (2005). Delaying the empiric treatment of candida bloodstream infection until positive blood culture results are obtained: a potential risk factor for hospital mortality. Antimicrob Agents Chemother.

[35] Chen Z, Yu J, Song Y, Chui D (2010). Aging Beijing: challenges and strategies of health care for the elderly. Ageing Res Rev.

[36] Blot SI, Vandewoude KH, Hoste EA, Colardyn FA (2002). Effects of nosocomial candidemia on outcomes of critically ill patients. Am J Med.

[37] Swoboda SM, Merz WG, Lipsetta PA (2003). Candidemia: the impact of antifungal prophylaxis in a surgical intensive care unit. Surg Infect (Larchmt).

[38] Andes DR, Safdar N, Baddley JW, Playford G, Reboli AC, Rex JH, Sobel JD, Pappas PG, Kullberg BJ (2012). Impact of treatment strategy on outcomes in patients with candidemia and other forms of invasive candidiasis: a patient-level quantitative review of randomized trials. Clin Infect Dis.

[39] Luzzati R, Cavinato S, Deiana ML, Rosin C, Maurel C, Borelli M: Epidemiology and outcome of nosocomial candidemia in elderly patients admitted prevalently in medical wards. *Aging Clin Exp Res* 2014 [Epub ahead of print]. 10.1007/s40520-014-0251-x 24923673

[40] Liu CY, Huang LJ, Wang WS, Chen TL, Yen CC, Yang MH, Hsiao LT, Chen PM, Chiou TJ (2009). Candidemia in cancer patients: impact of early removal of non-tunneled central venous catheters on outcome. J Infect.

